# Avian and Human Influenza A Virus Receptors in Bovine Mammary Gland

**DOI:** 10.3201/eid3009.240696

**Published:** 2024-09

**Authors:** Charlotte Kristensen, Henrik E. Jensen, Ramona Trebbien, Richard J. Webby, Lars E. Larsen

**Affiliations:** University of Copenhagen, Frederiksberg, Denmark (C. Kristensen, H.E. Jensen, L.E. Larsen);; Statens Serum Institut, Copenhagen, Denmark (R. Trebbien);; St. Jude Children’s Research Hospital, Memphis, Tennessee, USA (R.J. Webby)

**Keywords:** influenza A virus, influenza, viruses, host tropism, sialic acid, receptor, cattle, United States

## Abstract

An outbreak of influenza A (H5N1) virus was detected in dairy cows in the United States. We detected influenza A virus sialic acid -α2,3/α2,6-galactose host receptors in bovine mammary glands by lectin histochemistry. Our results provide a rationale for the high levels of H5N1 virus in milk from infected cows.

Influenza A virus (IAV) is a negative, single-stranded RNA virus. Viral evolution has enabled some IAVs to cross species barriers and to be established in humans and various mammals (*1*). Cattle are susceptible to infection with influenza C and D viruses but have been regarded as almost resistant to infection with IAV (*2*). An unexpected highly pathogenic avian influenza (HPAI) virus H5N1 (clade 2.3.4.4b) was detected in dairy cattle in Texas, USA, and has spread to 131 herds in 12 different states in the United States (*3*,*4*). Although extremely high levels of virus in milk from infected cows (*5*) were unexpected, previous studies and a report from Friedrich-Loeffler Institute (FLI; Insel Riems, Germany) (*6*) have shown that the inoculation of IAVs into the mammary glands of cows and goats results in productive viral infection (*2*).

Hemagglutinin (HA) binds to sialic acids (SA) terminally attached to glycans, enabling viral endocytosis and membrane fusion. Hemagglutinins of human- and swine-adapted IAVs frequently prefer SAs linked to galactose (Gal) in an α2,6 linkage (SA-α2,6, human receptor), whereas avian IAVs prefer an α2,3 linkage (SA-α2,3, avian receptor) (*7*). Furthermore, IAVs adapted to chickens generally prefer SA-α2,3-Gal with a β1,4 linkage to N-acetylgalactosamine (GalNac; SA-α2,3-Gal-β1,4-GalNac, referred to as chicken receptor), whereas IAVs isolated from ducks favor SA-α2,3-Gal with a β1,3 linkage to N-acetylglucosamine (GlcNac; SA-α2,3-Gal-β1,3-GlcNac, referred to as duck receptor) (*7*).

To investigate IAV receptor expression on the surface of epithelial cells, in situ techniques, such as lectin histochemistry, are useful (*8*). A limitation of using lectins is that they provide information only about the terminal end of the host receptors; a complete quantification of the receptors is not possible. Three studies have reported the IAV receptors in the bovine respiratory tract by using lectins (*9*–*11*), but studies describing the IAV receptor distribution in other bovine tissues are sparse. The aim of this study was to investigate the in situ expression of IAV receptors in the bovine mammary glands by lectin histochemistry.

## The Study

We included 2 archived bovine mammary glands obtained from the same lactating dairy cow (4 years of age) of a Danish Holstein breed and included mammary glands from 8 cows of different breeds and ages obtained from a slaughterhouse in Denmark (Table). The tissues were formalin-fixed, paraffin-embedded, and cut into 4–5 µm sections.

**Table:** The average percentage of epithelial staining of the human and duck influenza A virus receptors in the alveoli and ducts in 9 cows with different breeds, ages, and parity status*.

Breed	Age, y	Parity	Lactation†	% SNA		% MAA-II, alveoli‡
				Alveoli	Ducts	
Jersey	6	4	Late	53	18	37
Danish Holstein	4	2	Late	57	3	47
	7	5	Late	40	21	46
	6	4	Early/mid	47	4	44
	5	2	Late	58	24	61
	6	4	Early/mid	51	10	49
	5	3	Early/mid	55	1	51
	4	3	Late	52	32	48
Unknown	Unknown	Unknown	Early/mid	59	6	47
Average	5	3	NA	54	13	48

*MAA-II, duck receptor; NA, not applicable; SNA, human receptor. †All images were obtained from active alveoli of the mammary glands and the staining is therefore not representative for the whole section of cows in late lactation. The lactation period was determined by investigating the luminal size of the alveoli and the amount of connective tissue (*12*). ‡The staining did not exceed the amount of staining observed with the neuraminidase treatment, suggesting a nonspecific staining in the ducts.

**Figure 1:**
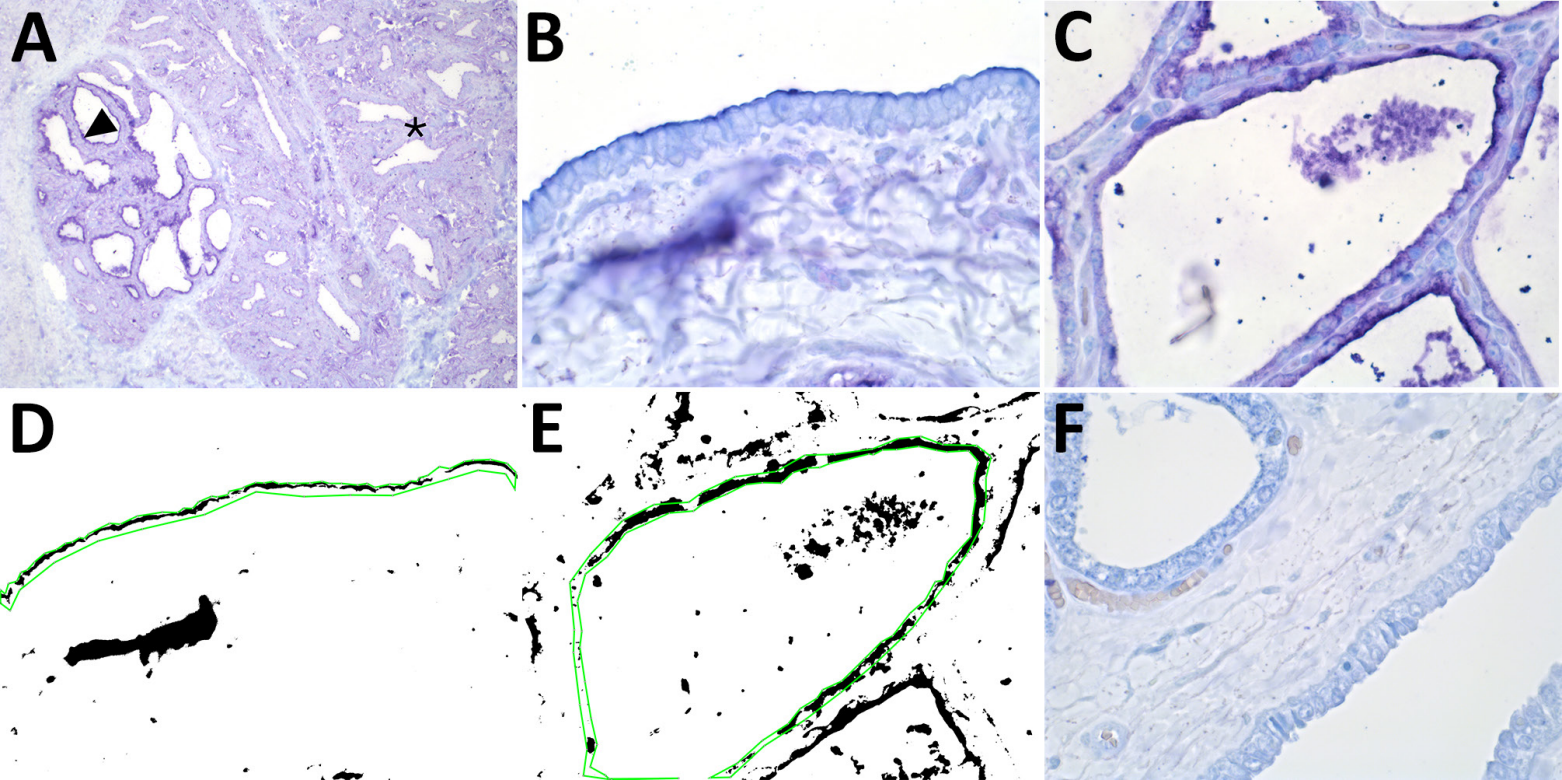
Results of staining showing wide expression of the human receptor for influenza A virus (designated SNA) in the bovine mammary gland. A) An example of the SNA staining of a mammary gland from a cow in late lactation. Arrowhead indicates expression of the human receptor in the active alveoli. Asterisks indicate less staining in the less active alveoli. Original magnification ×10. B, C) SNA staining of a duct (B) and (C) an alveolus in a 7-year-old cow. Original magnification ×60. D, E) Positive staining obtained from the image analysis. Green line shows the region of interest. Original magnification ×60. F) A neuraminidase pretreatment negative control showed markedly reduced staining of the SNA lectin. Original magnification ×60. The staining was visualized using Vector Blue (Vector Laboratories, https://vectorlabs.com).

**Figure 2:**
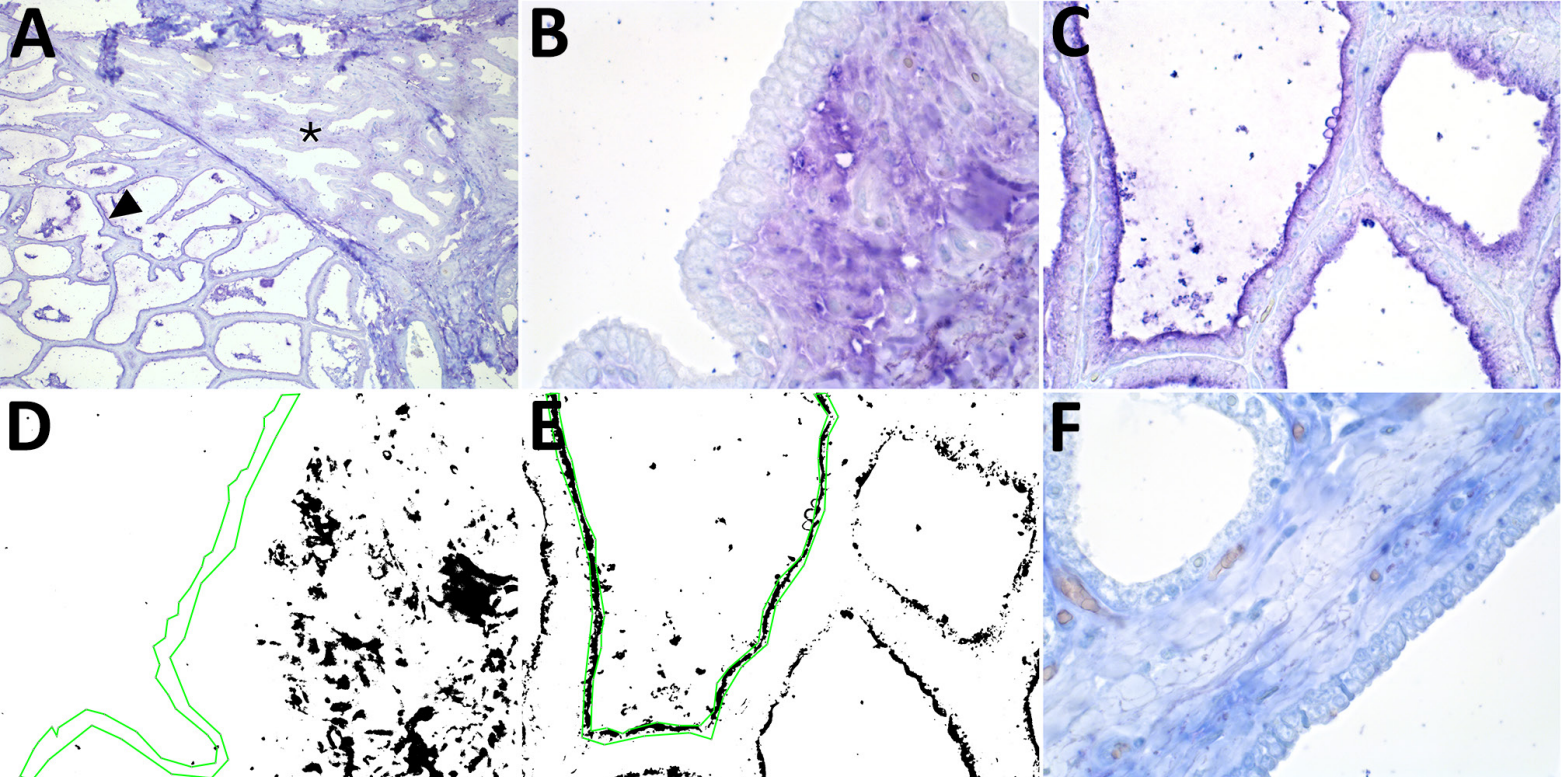
Results of staining showing wide expression of the duck receptor for influenza A virus (designated MAA-II) in the alveoli of the bovine mammary gland. A) An example of the MAA-II staining of a mammary gland from a cow in late lactation. Arrowhead indicates expression of the duck receptor in active alveoli. Asterisks indicate less staining in the less active alveoli. Original magnification ×10. B, C) MAA-II staining of a duct (B) and an alveolus (C) in a 3-year-old cow. Original magnification ×60. D, E) Positive staining obtained from the image analysis. Green line shows the region of interest. Original magnification ×60. F) A neuraminidase pretreatment negative control showed markedly reduced staining of the MAA-II lectin in the alveolus but some nonspecific staining in the duct epithelium. The staining was visualized using Vector Blue (Vector Laboratories, https://vectorlabs.com). Original magnification ×60.

We detected the human receptor SA-α2,6 using biotinylated *Sambucus nigra* lectin (SNA) (B-1305-2; Vector Laboratories, https://vectorlabs.com). We detected the chicken receptor SA-α2,3-Gal-β1,4 using biotinylated *Maackia amurensis* lectin I (MAA-I) (B-1315-2; Vector Laboratories) and the duck receptor (SA-α2,3-Gal-β1,3) biotinylated MAA-II (B-1265-1; Vector Laboratories) as previously described (*13*). We semiquantified the staining on the surface of the epithelial cells as the percentage of positive surface area in 2 images from each slide (Figure 1, panels D, E; Figure 2, panels D, E) and reported the average (*13*). We applied 2 negative controls. To investigate potential background staining (blind), we added no lectins; to investigate the amount of nonspecific binding, we applied a neuraminidase pretreatment before each lectin, as previously reported (*13*). We included a confirmed IAV-negative pig lung as a positive control.

**Figure 3:**
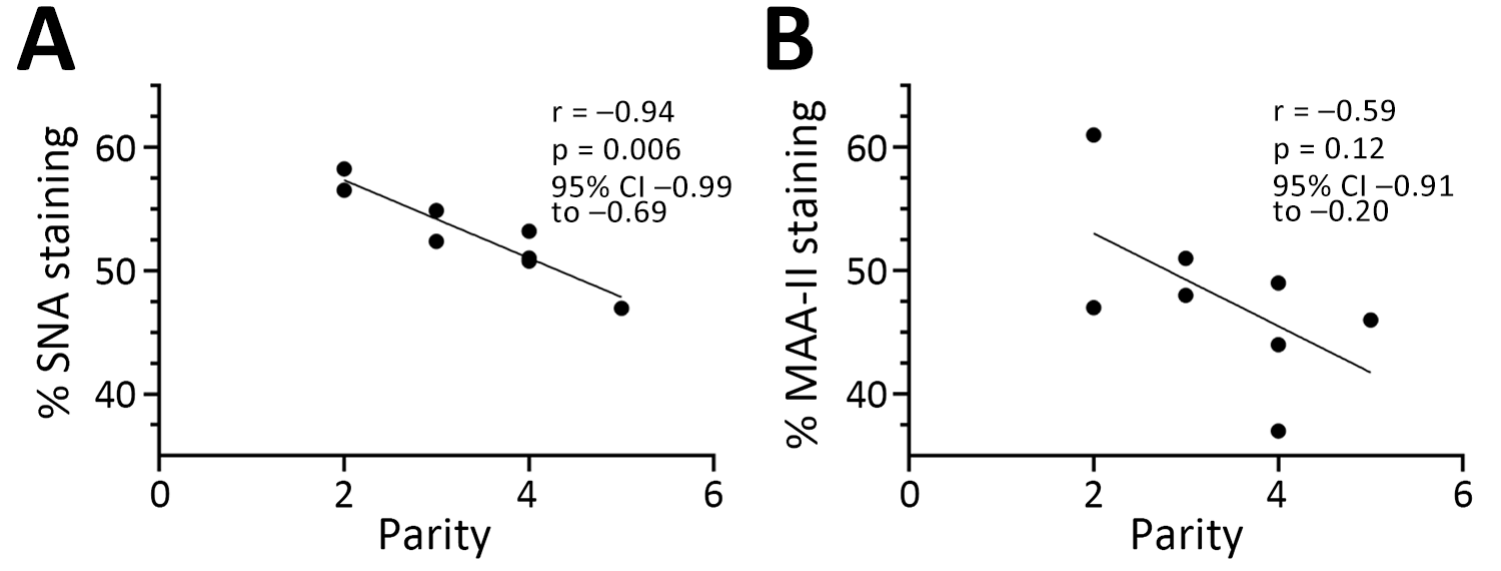
Pearson correlation coefficient showing a significant negative correlation between the percentage of SNA staining (bovine receptor) in the alveoli and the parity status of the cows (A) but not for MAA-II staining (duck receptor) (B). Original magnification ×60. Statistics and graphs made using GraphPad Prism version 10.2.3 (GraphPad Software, www.graphpad.com).

In the mammary gland, the human receptor (detected by SNA) and the duck receptor (detected by MAA-II) were widely distributed in the alveoli, but less so in the ducts, whereas we detected no positive staining of the chicken receptor by MAA-I (Table; Figure 1; Figure 2; Appendix Tables), except for 1 cow expressing the chicken receptor in a lactiferous duct. Both human and duck receptors were primarily expressed in the active alveoli and less expressed in less active alveoli (Figure 1; Figure 2). We found a significant negative correlation between the percentage of SNA staining in the alveoli and the parity status of the cows but not with the MAA-II lectin staining (Figure 3).

We detected nonspecific staining (positive staining after neuraminidase pretreatment) in the endothelial cells with the SNA and MAA-I lectins, in the bovine erythrocytes with the MAA-I lectin, and in the ducts of the MAA-II lectin, but observed no nonspecific staining elsewhere (Appendix Figure). The positive controls of the pig lung corresponded to previous findings (*13*) (Appendix Figure).

Our investigation evaluated the expression of IAV receptors in situ in the mammary gland of cattle, which typically has been considered less susceptible to IAV infection (*2*). Of note was the finding that both the human receptors (SA-α2,6) and the duck receptors (SA-α2,3-Gal-β1,3) were highly expressed in the active alveoli in mammary glands but lower expressed in the less active alveoli, which could indicate that cows peaking in lactation are more susceptible for infection. The findings of the 2 receptors are in agreement with 2 novel studies investigating the IAV receptors in the bovine mammary gland of 2–3 cows (*11*; M.R. Carrasco et al., unpub. data, https://doi.org/10.1101/2024.05.24.595667). The transmission routes and the pathogenesis of H5N1 in cows remain unclear, but the virus remains infectious after 1 hour on the milking equipment (V.L. Sage et al., unpub. data, https://doi.org/10.1101/2024.05.22.24307745); the US Department of Agriculture has reported that only some udder quarters may be involved in infection, suggesting an ascending infection as a possible transmission route. Of interest, the human and duck receptors were less expressed in the ducts of the mammary gland, making an ascending mammary gland infection using those receptors more challenging. An investigation of the receptor binding preference of A/Texas/37/2024 showed that an I199T mutation of the hemagglutinin protein increased the receptor binding breadth to a higher number of conformations of the avian receptor, including hybrid N-glycans, compared with previous H5N1 viruses (M.R. Good et al., unpub. data, https://doi.org/10.1101/2024.06.22.600211); those hybrid N-glycans might not be detected by the lectins used in our study (*14*). M.R. Carrasco, et al. (unpub. data) showed limited binding of older AIV strains and a mouse-adapted human influenza strain (PR8) in the mammary gland of cows, whereas the preliminary report from FLI (*6*) showed that genotypes from Europe of the clade 2.3.4.4b H5 HPAI viruses could also replicate in the mammary glands of cows. Thus, more studies are needed to investigate the susceptibility of cows to different IAV strains and variants.

The SNA lectin detects both of the 2 most common sialic acids, N-acetylneuraminic acid (Neu5Ac) and N-glycolylneuraminic acid (Neu5Gc) (*14*). Neu5Ac is mainly the preferred SA for IAVs, except for equine IAVs, which have a higher preference for Neu5Gc (*7*). Cattle express both Neu5Ac and Neu5Gc in their tissues, but the amount of Neu5Ac in bovine milk is 30 times higher than for Neu5Gc (*15*), which indicates that the SNA staining detected in our study was caused by staining of Neu5Ac. However, further studies such as mass spectrometry, which gives detailed information about the IAV receptors (e.g., sialic acid type, length, branching), are needed to confirm the cause of SNA staining and also to accomplish a comprehensive quantification of the distribution of receptors in bovine tissues (*8*).

## Conclusions

The expression of the duck receptor in the mammary gland of cows fits with the observed widespread infections among cattle in the United States with HPAI H5N1. The co-expression of both human and avian receptors in the mammary glands indicates susceptibility to other IAVs than those from avian origin, which is worrying from a zoonotic perspective. The presence of the IAV receptors, however, does not provide evidence that cattle are susceptible to all IAVs; other host factors (*1*) probably play a role for successful replication.

Appendix Additional information about avian and human influenza A virus receptors in bovine mammary glands.

## References

[1] Yoon S-W, Webby RJ, Webster RG. Evolution and ecology of influenza A viruses. In: Compans R, Oldstone M, editors. Influenza pathogenesis and control. Vol. I. Current topics in microbiology and immunology. Cham (Switzerland); Springer; 2014. p 359–75. 10.1007/82_2014_396 24990620

[2] Sreenivasan CC, Thomas M, Kaushik RS, Wang D, Li F. Influenza A in bovine species: a narrative literature review. Viruses. 2019;11:561. 10.3390/v11060561 31213032 PMC6631717

[3] Centers for Disease Control and Prevention. H5N1 bird flu: current situation summary. [cited 2024 Jun 28]. https://www.cdc.gov/flu/avianflu/avian-flu-summary.htm

[4] Ly H. Highly pathogenic avian influenza H5N1 virus infections of dairy cattle and livestock handlers in the United States of America. Virulence. 2024;15:2343931. 10.1080/21505594.2024.2343931 38632687 PMC11028003

[5] World Health Organization. Joint FAO/WHO/WOAH preliminary assessment of recent influenza A(H5N1) viruses. 23 April 2024 [cited 2024 Jun 24]. https://www.who.int/publications/m/item/joint-fao-who-woah-preliminary-assessment-of-recent-influenza-a(h5n1)-viruses

[6] Friedrich-Loeffler-Institut. Rapid risk assessment for highly pathogenic avian influenza H5 (HPAI H5) clade 2.3.4.4b. 2024 [cited 2024 Jul 15]. https://www.openagrar.de/servlets/MCRFileNodeServlet/openagrar_derivate_00060240/FLI-Risikoeinschaetzung_HPAI_H5_2024-07-05_en.pdf

[7] Zhao C, Pu J. Influence of host sialic acid receptors structure on the host specificity of influenza viruses. Viruses. 2022;14:2141. 10.3390/v14102141 36298694 PMC9608321

[8] Varki NM, Varki A. Diversity in cell surface sialic acid presentations: implications for biology and disease. Lab Invest. 2007;87:851–7. 10.1038/labinvest.3700656 17632542 PMC7100186

[9] Thontiravong A, Rung-ruangkijkrai T, Kitikoon P, Oraveerakul K, Poovorawan Y. Influenza A virus receptor identification in the respiratory tract of quail, pig, cow and swamp buffalo. Wetchasan Sattawaphaet. 2011;41:15. 10.56808/2985-1130.2322

[10] Uprety T, Sreenivasan CC, Bhattarai S, Wang D, Kaushik RS, Li F. Isolation and development of bovine primary respiratory cells as model to study influenza D virus infection. Virology. 2021;559:89–99. 10.1016/j.virol.2021.04.003 33862336 PMC8205979

[11] Nelli RK, Harm TA, Siepker C, Groeltz-Thrush JM, Jones B, Twu NC, et al. Sialic acid receptor specificity in mammary gland of dairy cattle infected with highly pathogenic avian influenza A(H5N1) virus. Emerg Infect Dis. 2024;30:1361–73. 10.3201/eid3007.240689 38861554 PMC11210646

[12] Hurley WL, Loor JJ. Mammary gland: growth, development and involution. In: Fuquay JW, Fox PF, McSweeney PLH, editors. Encyclopedia of dairy sciences. 2nd ed. Vol. 3. San Diego: Academic Press; 2011. p. 338–345.

[13] Kristensen C, Larsen LE, Trebbien R, Jensen HE. The avian influenza A virus receptor SA-α2,3-Gal is expressed in the porcine nasal mucosa sustaining the pig as a mixing vessel for new influenza viruses. Virus Res. 2024;340:199304. 10.1016/j.virusres.2023.199304 38142890 PMC10793167

[14] Bojar D, Meche L, Meng G, Eng W, Smith DF, Cummings RD, et al. A useful guide to lectin binding: machine-learning directed annotation of 57 unique lectin specificities. [PubMed]. ACS Chem Biol. 2022;17:2993–3012. 10.1021/acschembio.1c00689 35084820 PMC9679999

[15] Spichtig V, Michaud J, Austin S. Determination of sialic acids in milks and milk-based products. Anal Biochem. 2010;405:28–40. 10.1016/j.ab.2010.06.010 20553868

