# Supplementary material for: Avian and Human Influenza A Virus Receptors in Bovine Mammary Gland
Source: Emerg Infect Dis. 2024 Sep;30(9):1907–11. doi: 10.3201/eid3009.240696 (PMC11347012; doi:10.3201/eid3009.240696)
Supplement: Appendix — Additional information about avian and human influenza A virus receptors in bovine mammary glands. [file 24-0696-Techapp-s1.pdf]

Article DOI: http://doi.org/10.3201/eid3009.240696

EID cannot ensure accessibility for supplementary materials supplied by authors. Readers who have difficulty accessing supplementary content should contact the authors for assistance.

Avian and Human Influenza A Virus Receptors in Bovine Mammary Gland

Appendix

Appendix Table 1. Raw measurements from the semi quantification of the MAA-II staining (duck receptor) performed in Image J. Measurements from two images of the alveoli and ducts from each cow and the calculated average are reported. Two mammary glands were examined from cow number 9.

			Mammary gland positive staining in %						
Cow no.	Age	Parity	Alveoli 1	Alveoli 2	Average	Duct 1	Duct 2	Average	Threshold
1	6	4	16.8	56.9	37	2.1	5	4	180
2	-	-	42.6	51.4	47	1.5	0.2	1	182
3	4	2	52.5	41	47	1.9	0.1	1	165
4	7	5	55.1	36	46	11.3	7.7	10	174
5	6	4	38.3	50.5	44	0.9	2	1	175
6	5	2	64.2	57	61	0.7	0.3	1	189
7	6	4	50.4	47.6	49	3.9	19.8	12	173
8	5	3	45.7	56	51	1.3	3	2	159
9a	4	3	57.3	41.3	49	0	1.1	1	186
9b	4	3	52.8	41	47	0.4	7.3	4	188
9average	4	3	55.05	41.15	48	0.2	4.2	2	-
**Average**	5	3	46	48	**48**	2	5	**4**	**177.1**

Appendix Table 2. Raw measurements from the semi quantification of the SNA staining (human receptor) performed in Image J. Measurements from two images of the alveoli and ducts from each cow and the calculated average are reported. Two mammary glands were examined from cow number 9.

			Mammary gland positive staining in %						
Cow no.	Age	Parity	Alveoli 1	Alveoli 2	Average	Duct 1	Duct 2	Average	Threshold
1	6	4	43.2	63.2	53	29.1	6.3	17.7	151
2	-	-	78.8	39.7	59	8.9	3.1	6	134
3	4	2	57.7	55.4	57	0.1	5.2	2.65	130
4	7	5	54.2	39.7	47	30.8	10.6	20.7	153
5	6	4	59.3	42.3	51	6.2	1.6	3.9	163
6	5	2	56.3	60.2	58	7.2	40.9	24.05	133
7	6	4	65.1	37	51	2.6	17.7	10.15	140
8	5	3	68.8	41	55	1	0.6	0.8	127
9a	4	3	64.4	36.7	51	49.5	16.5	33	178
9b	4	3	49.2	59.4	54	18.5	41.3	29.9	167
9average	4	3	56.8	48.05	52	34	28.9	31.45	-
**Average**	5	3	60	47	**54**	11	11	**13**	**147.6**

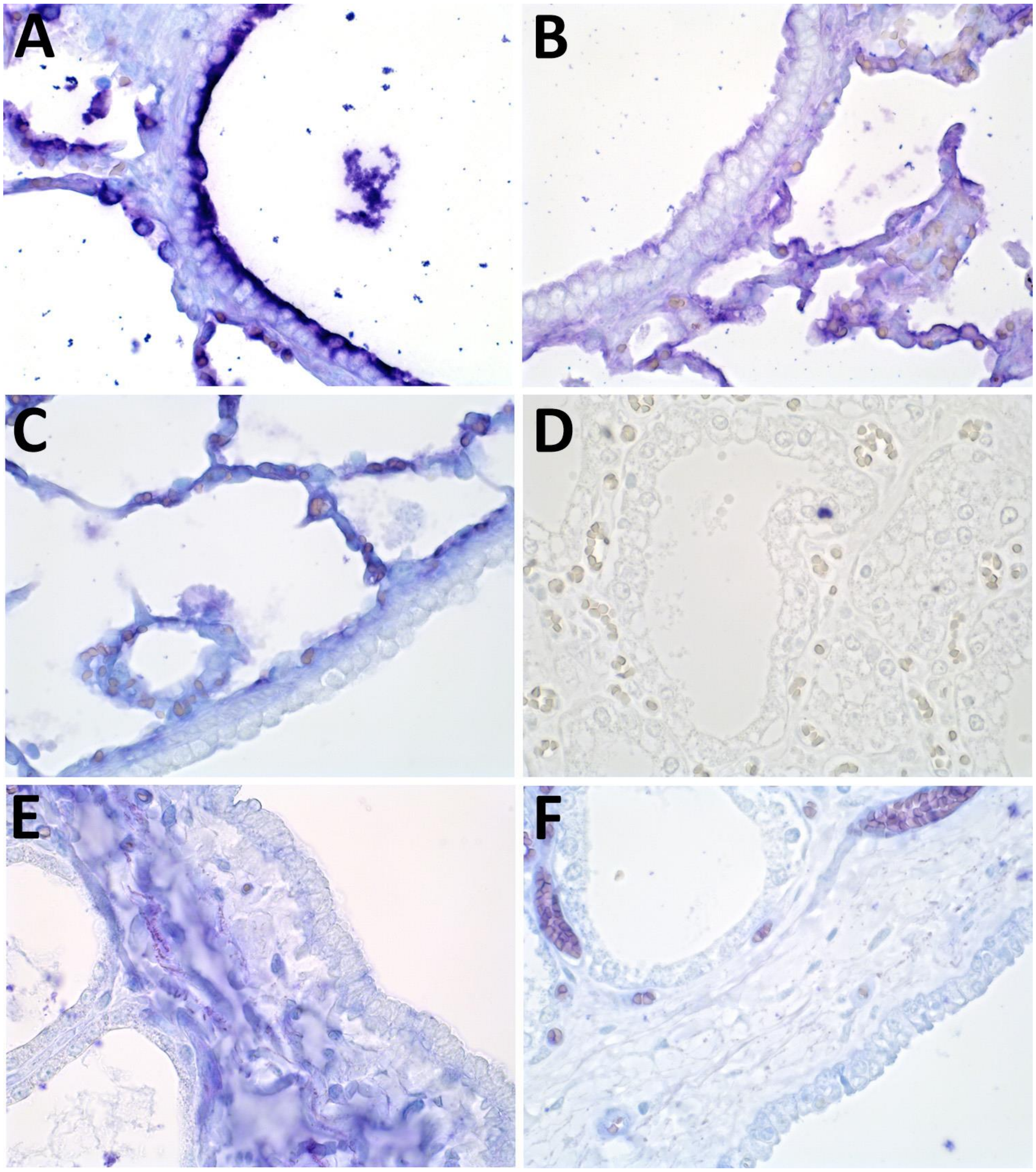

Appendix Figure. Positive and negative controls from the lectin histochemistry showed no nonspecific staining of the epithelial cells. A) The positive control was performed on a porcine lung, this is an example of the A) SNA, B) MAA-II, and C) MAA-I staining. D) A slide of a bovine mammary gland with no lectin added showing no background signal. E) MAA-I showed no positive staining of the epithelial cells in the bovine mammary gland. F) Neuraminidase controls of the MAA-I staining showed a non-specific staining of the bovine erythrocytes. The staining was visualized by Vector Blue.
